# What drives wild pig (*Sus scrofa*) movement in bottomland and upland forests?

**DOI:** 10.1186/s40462-024-00472-y

**Published:** 2024-04-25

**Authors:** Tyler S. Evans, Natasha Ellison, Melanie R. Boudreau, Bronson K. Strickland, Garrett M. Street, Raymond B. Iglay

**Affiliations:** Department of Wildlife, Fisheries and Aquaculture, Mississippi State University, 775 Stone Boulevard, Mississippi State, Mississippi, 39762 USA

**Keywords:** Anthropogenic, Exotic species, Invasion, Resource selection, Space use, Spatiotemporal

## Abstract

**Background:**

The wild pig (*Sus scrofa*) is an exotic species that has been present in the southeastern United States for centuries yet continues to expand into new areas dominated by bottomland and upland forests, the latter of which are less commonly associated with wild pigs. Here, we aimed to investigate wild pig movement and space use attributes typically used to guide wild pig management among multiple spatiotemporal scales. Our investigation focused on a newly invaded landscape dominated by bottomland and upland forests.

**Methods:**

We examined (1) core and total space use using an autocorrelated kernel density estimator; (2) resource selection patterns and hot spots of space use in relation to various landscape features using step-selection analysis; and (3) daily and hourly differences in movement patterns between non-hunting and hunting seasons using generalized additive mixed models.

**Results:**

Estimates of total space use among wild pigs (*n* = 9) were smaller at calculated core (1.2 ± 0.3 km^2^) and 90% (5.2 ± 1.5 km^2^) isopleths than estimates reported in other landscapes in the southeastern United States, suggesting that wild pigs were able to meet foraging, cover, and thermoregulatory needs within smaller areas. Generally, wild pigs selected areas closer to herbaceous, woody wetlands, fields, and perennial streams, creating corridors of use along these features. However, selection strength varied among individuals, reinforcing the generalist, adaptive nature of wild pigs. Wild pigs also showed a tendency to increase movement from fall to winter, possibly paralleling increases in hard mast availability. During this time, there were also increases in anthropogenic pressures (e.g. hunting), causing movements to become less diurnal as pressure increased.

**Conclusions:**

Our work demonstrates that movement patterns by exotic generalists must be understood across individuals, the breadth of landscapes they can invade, and multiple spatiotemporal scales. This improved understanding will better inform management strategies focused on curbing emerging invasions in novel landscapes, while also protecting native natural resources.

**Supplementary Information:**

The online version contains supplementary material available at 10.1186/s40462-024-00472-y.

## Background

Whether deliberate or accidental, human activities continue to introduce or support exotic species in previously unaffected regions [1, 2]. Once introduced, exotic species can naturalize and expand when habitat requirements are met and they maintain a sustainable population [3]. Improving our understanding of how these species move (e.g. spread or disperse [4–6]), establish home ranges [7, 8], and select resources [8–10] over various spatiotemporal scales in novel, previously uninvaded landscapes may aid in informing control efforts [8] and identifying at-risk native species, communities, or ecosystems of concern [11]. Space use of exotic species often leads to detrimental effects on biotic (e.g. native plants, animals, invertebrates [12, 13]) and abiotic (e.g. soil, nutrients, water [14, 15]) ecosystem components, which collectively provide myriad ecosystem services (e.g. wetland ecosystems providing flood abatement, water quality improvement, and capturing and neutralizing pollutants [16]) that are often taken for granted. The movements of exotic species may also facilitate colonization and spread of other exotic species (i.e. plants) through zoochory, representing another concern for local ecosystems [17].

Advances in GPS technology continue to increase spatial accuracy and temporal resolution of data, allowing for the investigation of animal movement and space use among multiple spatiotemporal scales. Across larger spatiotemporal scales, animal movement patterns can translate into measures of total space use (e.g. home range [18]), a metric that can help elucidate the scale at which management or control should be implemented [19, 20]. At finer spatial scales (as defined in [21]), investigating resource selection can further differentiate the use of various land coverages by a species and inform a species’ life history requirements or preferences, which may include foraging, bedding, and nesting sites [22–24]. Finally, our ability to identify movement patterns at various temporal scales, such as those that occur in relation to seasonal fluctuations in environmental conditions or circadian patterns, can help determine what alters movement and space use in relation to various anthropogenic mechanisms such as hunting or trapping pressure (e.g. [25]) or biological rhythms [26]. Because drivers of space use may shift among spatiotemporal scales, these metrics hold value for successful management of a species, and the respective importance of each metric may become increasingly apparent when tasked with controlling exotic species [27].

The wild pig (*Sus scrofa*) is a successful exotic species that thrives in myriad landscapes, taking advantage of diverse food resources, water, and thermoregulatory cover [28, 29], and within much of its introduced range in the United States, represents a hybridization between domestic and wild individuals [30]. Wild pigs are capable of causing ecological damage by reducing understory vegetation and impacting habitat resources of native wildlife [31, 32]; disturbing soils [33], which also disrupts normal carbon cycling [34]; altering local hydrology and water quality [35]; and altering seedbanks [17]. Forest damage occurs through rubbing and rooting of trees [31, 36], and the consumption of hard mast sources affects tree regeneration in addition to reducing food resources for native fauna [37, 38]. Wild pig damage patterns can vary across a landscape as their movements shift in both space (i.e. from disturbed to undisturbed areas) and time (i.e. by hour of day or season) responding to various anthropogenic disturbances (e.g. hunting pressure [39]; roads with varying traffic intensity [40]). Streams and other water bodies have been positively associated with wild pig space use as well as forests, wetlands, and low elevations [41–43] as these areas often contain landcover features (i.e. water and cover) that enable thermoregulation. Within these areas, wild pigs often exhibit site fidelity associated with specific natural landscape features during specific times of day (e.g. diurnal and crepuscular use of forest and water bodies, respectively [41]). High site fidelity and philopatry have also been associated with wild pigs among all sex and age classes, with a strong seasonal effect (e.g. greatest in winter and spring [44]).

While wild pig space use has been intensively investigated in agricultural-forested matrices [45, 46], grassland-shrubland dominated landscapes [47], and grassland-forested matrices [48, 49], little is known about how wild pigs might use large tracts of bottomland and upland forests in North America. Therefore, to improve our understanding of new wild pig invasions of contiguously forested landscapes and inform management and control actions, we (1) estimated the size of core and total utilization distributions; (2) quantified selection patterns in relation to various landscape features; (3) identified hot spots of space use within utilization distributions; and (4) identified daily and hourly differences in movement patterns between non-hunting and hunting seasons. Operating under the hypothesis that wild pigs’ overall space use would reflect the species’ tendency for philopatry and site fidelity, we predicted that average utilization distribution size would be smaller than what has been reported in other studies of wild pigs located in less thermoregulatory-hospitable landscapes (e.g. grassland-shrubland [47]), yet larger than estimates reported in potentially more thermoregulatory-hospitable areas (e.g. coastal marshlands [50]). Second, considering the physiological need for wild pigs to thermoregulate using external sources, we predicted wild pigs would select areas associated with water (e.g. wetlands and streams), relative to landscape features with less perceived thermoregulatory value (e.g. upland forest, shrubland, human development). These selection patterns would also manifest in the form of heterogeneities in the intensity of use within utilization distributions as specified features are also heterogeneous across a landscape. Finally, operating under the hypothesis that anthropogenic pressures drive wild pig movements across spatiotemporal gradients, we expected wild pigs would have shorter daily movement distances and greater crepuscular or nocturnal movement when anthropogenic pressure was greatest.

## Methods

### Study site

While the earliest reports of wild pigs in the southeastern United States date back to 1539 [51], their distribution is increasing. For example, in Mississippi wild pig occurrences increased in coverage from 4 to 38% of the state’s total land area between 1988 and 2009 [52]. Investigations of wild pig space use in Mississippi have focused on movement behaviors in captive wild pigs [53], movements and survival using VHF telemetry and imagery [54], spatiotemporal movements in coastal areas [50], and resource selection in vastly contrasting landscapes (e.g. Mississippi Alluvial Valley [46]).

Comprised of 19,425 hectares of bottomland and upland forest, the Sam D. Hamilton Noxubee National Wildlife Refuge (NNWR) was established in 1940 as a refuge and breeding ground for migratory birds and other wildlife, and has been conserved, managed, and as necessary, restored for the benefit of fish, wildlife, plant resources, and humans [55]. Bottomland forests were seasonally-flooded, closed canopy areas with forest canopies dominated by (*Quercus* spp.) and hickory (*Carya* spp.) species complemented by midstory tree species common throughout the southeastern United States such as American hornbeam (*Carpinus caroliniana*), ash (*Fraxinus* spp.), gums (*Liquidambar styraciflua* and *Nyssa sylvatica*), elms (*Ulmus* spp.), maples (*Acer* spp.), and sugarberry (*Celtis laevigata*). Upland forests were either frequently burned and relatively open loblolly pine (*Pinus taeda*) stands with herbaceous understories, loblolly pine stands with established woody midstories, or mixed pine-hardwood stands, especially along ridges and transition zones from upland to bottomland areas. In addition to its value for waterfowl, a variety of common native game species (e.g. white-tailed deer, *Odocoileus virginianus*) and several avian species of concern (e.g. red-cockaded woodpecker, *Leuconotopicus borealis*) use the NNWR. Observations of wild pigs and associated disturbances on the NNWR have become increasingly noticeable since 2014 despite their longstanding Mississippi residency (Taylor Hackemack, United States Fish and Wildlife Service, pers. comm.). As wild pigs have an early age of sexual maturity (5–8 months) and high reproductive capacity (3–11 piglets across 1–2 litters/year), population growth likely cannot be controlled without a substantive annual reduction (e.g. 70% [56, 57]) achieved through coordinated control measures (i.e. trapping, aerial gunning), particularly in areas with high resource availability that support larger populations (e.g. bottomland forests with hard mast [58]). While there have never been substantive control efforts implemented on the NNWR, investigations into space use of emergent invasions such as these can inform future control efforts on the NNWR and other areas with similar landscapes experiencing new invasions. White-tailed deer hunting seasons on the NNWR (with concurrent wild pig hunting opportunities) occurred during fall with archery-based hunting between 1 October – 19 November and firearm-based hunting between 20 November – 31 December. According to white-tailed deer harvest reports for Mississippi, there is a difference in hunting pressure across types of hunting as firearm hunters harvest 3x more white-tailed deer than archery hunters. While hunting access is only permitted during daylight hours on the NNWR, hunters targeting other game species are also known to kill wild pigs if they encounter them opportunistically. Dogs or bait are not permitted for hunting purposes on the NNWR.

### Trapping, handling, and GPS collar deployment

Between November 2020 and September 2021, we trapped unique partial sounders on the NNWR using a HogEye camera (Wildlife Dominion Management LLC, Mississippi, USA), dual-gated panel trap (Big Pig, Backwoods Solutions LLC, Mississippi, USA), and suspended corral trap (Boar Buster, Noble Research Institute LLC, Oklahoma, USA). Within each partial sounder, an adult female (*n* = 10; mean: 68.9 ± 5.2 kg) was chosen for immobilization. Before immobilization, all wild pigs in the trap, other than the chosen female were euthanized via gunshot to the head. Once all other wild pigs were euthanized, the chosen female was immobilized using an intramuscular injection of Medetomidine HCl (0.06 mg/kg), Midazolam HCl (0.3 mg/kg), and Butorphanol Tartrate (0.3 mg/kg; ZooPharm Inc., Wyoming, USA [59]). After immobilization, each female was fitted with a GPS collar (Vectronic-Aerospace Iridium, Berlin, Germany) programmed to collect relocations at a 2-h fix rate and transmit locations to an online server daily (i.e. locations could be remotely downloaded from satellite transmissions). Each female also received two livestock-grade ear tags (Y-TEX, Wyoming, USA). Immobilization was reversed using Atipamezole (5.0 mg per 1.0 mg of Medetomidine). All trapping and handling protocols were in accordance with NNWR guidelines (United States Fish and Wildlife Service Permit #43620-20-013) and approved by Mississippi State University Institutional Animal Care and Use Committee (Protocol #IACUC-20-022).

### Analyses

A stationary collar test in various landcover types indicated that GPS collars were accurate to < 30 m and were only impacted in their collection in the densest of forest cover (5% loss of expected locations). We collected 12,970 GPS locations across wild pigs (375–2340 locations/female), each of which was assumed to represent a unique sounder. After ensuring locations reflected only those collected while active and on the wild pig (i.e. no locations after a collar was slipped by an animal), we cleaned GPS locations by censoring those (*n* = 5) that were clearly incorrect (i.e. improbable locations paired with nonsensical elevation readings). We also ensured that all retained locations followed a 2-h fix rate. Finally, we examined movement patterns of each wild pig to ensure individuals were moving independently. As two individuals with collars joined together for the duration of their collar deployment, we removed one wild pig from analyses to prevent pseudoreplication. This left 10,156 GPS locations across 9 individuals for use in analyses. Using these data, we analyzed (1) space use, (2) resource selection, and (3) temporal changes in movements. To examine overall space use, we first generated utilization distributions for each wild pig using an autocorrelated kernel density estimator [60] at two isopleths: (1) 90%, which we used to represent a measure of total space use [61]; and (2) a measure of core space use that we calculated for each wild pig using a threshold value beyond which the estimated area increased at a rate greater than the probability of use [62]. Autocorrelated kernel density estimates were created using the *ctmm* R package [63] in R v. 4.1.0 [64].

**Fig. 1:**
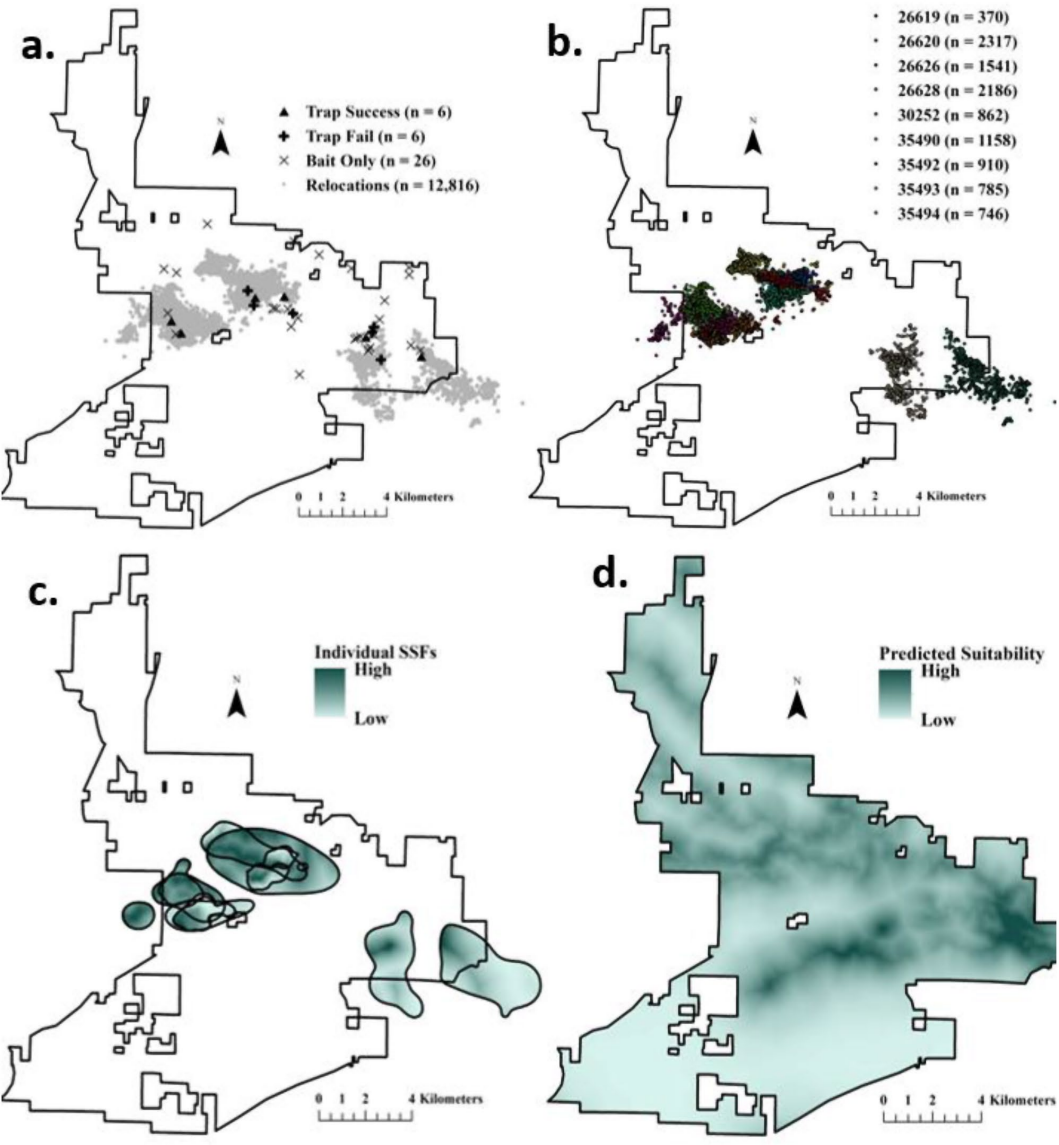
Relocations of adult female wild pigs (*Sus scrofa*; *n* = 9) relative to successful trap locations, failed trap locations, and bait-only locations in the Sam D. Hamilton Noxubee National Wildlife Refuge in Mississippi, USA (**a**), individual relocations (**b**), individual step-selection function hotspots (**c**), and predicted suitability given population-averaged beta coefficients (**d**)

To examine resource selection by wild pigs, we used step-selection analysis that models habitat selection relative to variation in local habitat availability [65]. Step-selection analyses assess resource selection by comparing each relocation (i.e. used) to plausible alternative relocations (i.e. available). For each used relocation, we generated 100 random available relocations by sampling step lengths (mean = 233.7 ± 2.6 m) from a parametric step length distribution (Additional File S1: Fig. 1) and turn angles from a uniform distribution given similar observed movement patterns among wild pigs. Step lengths were defined as straight-line distances between two successive fixes, while turn angles were the directional change in heading between successive steps. Thus, only movement bursts with ≥ 3 consecutive locations (i.e. over a minimum of 6 h) were included in our analysis to allow for the proper calculation of turn angles. For each used and available step, we extracted information related to 9 environmental covariates expected to be related to wild pig space use that included distances to various land cover and stream types. For land cover, we used the 2016 National Land Cover Database [66], and we reclassified the 14 land cover classes present on the NNWR into 7 classes including: water (open water and barren land), developed (open spaces, low and medium intensity development), shrub, field (hay/pasture and cultivated crops), herbaceous (herbaceous cover and herbaceous wetlands), woody wetland, and upland forest (deciduous-, evergreen-, and mixed-forest). Reclassifications were based either on known discrepancies between original classification and ground knowledge (e.g. barren class exclusively located in middle of two large lakes on NNWR) or perceived functional similarity of classes on the NNWR (e.g. deciduous, evergreen and mixed forests located in upland areas) relative to wild pig thermoregulatory constraints and concomitant decreases in foraging efficiency [28]. We transformed each land cover class into its own continuous variable by calculating Euclidean distance from each land cover type in ArcGIS [67]. Similarly, we calculated Euclidean distances (30-m resolution) to intermittent and perennial streams using shapefiles for each stream type [68]. All environmental covariates were centered and scaled prior to analysis.

We related used and available steps (1 and 0 as our response) to our environmental covariates using conditional logistic regression, with each stratum as the used and available locations at each timepoint. Because we encountered high individual variation in availability across individuals (i.e. relative to respective locations on the NNWR) but still desired to fit the same model across all wild pigs, we used a conditional logistic regression model with lasso regularization [69], using the *clogitL1* function from the *clogitL1* R package [70], as this allowed for elastic net penalization for model coefficients through use of a cross-validation procedure that provided a consistent method to identify an optimal model (i.e. containing beta-coefficient values at the minimum cross-validation statistic) for each wild pig. We exponentiated resulting model coefficients to calculate odds ratios and used these to generate maps of predicted selection intensity (i.e. risk of selecting a location based on landscape conditions) for each wild pig within its respective utilization distribution, and we also created a population average to solve the step-selection function over the entire NNWR landscape, although we acknowledge that a mismatch exists between the scale in the space being solved for, and the space within which availability was defined (i.e. the maximum step length). We generated each map using raster algebra in ArcGIS [67].

**Fig. 2:**
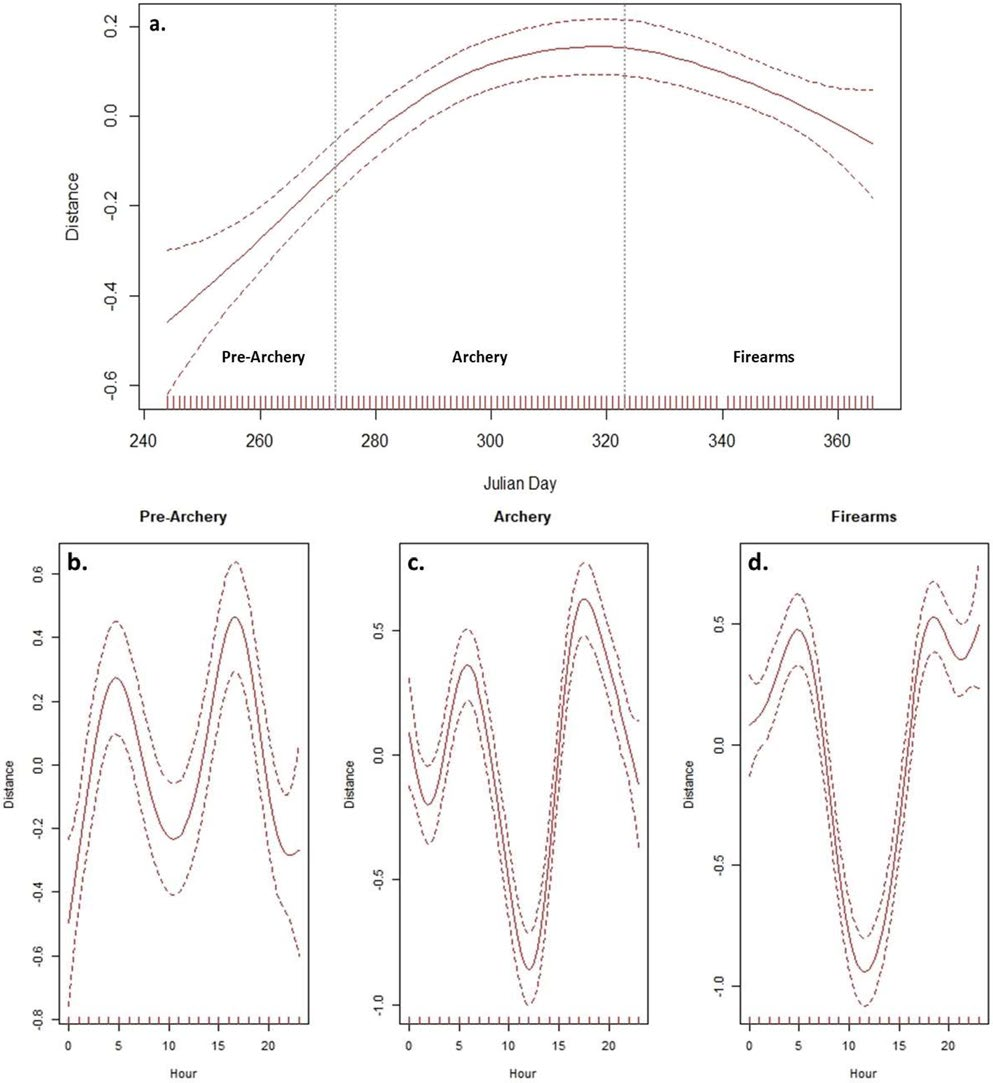
Generalized additive mixed model outputs representing daily distance traveled by adult female wild pigs (*Sus scrofa*; *n* = 9) trapped in the Sam D. Hamilton Noxubee National Wildlife Refuge (NNWR) in Mississippi, USA as a function of Julian day (**a**), and 2-h step length distance as a function of hour of day during the pre-archery (**b**), archery (**c**), and firearms (**d**) seasons

Finally, we investigated possible differences in seasonality of movements as these often relate to temporally dependent influences. To directly compare findings across individuals, we restricted our analyses to data collected between September and December as these months contained information from the greatest number of unique animals (Additional File S1: Fig. 2). Using only days with complete information (*n* = 12 relocations/day), we calculated average distances traveled by each wild pig for each Julian day across those months. In addition, given that wild pigs will change space use patterns in relation to anthropogenic disturbance [39, 71, 72], we also wanted to investigate differences in movement patterns relative to hunting pressure. Therefore, we calculated average distances traveled during each hour of the day within each part of the hunting season (pre-archery from 1 September – 30 September: *n* = 77 total days with complete information across wild pigs; archery season: *n* = 164 days; and firearms season: *n* = 151 days). We then examined average distance traveled in relation to Julian day and average distance traveled in relation to time of day in each season using generalized additive mixed models (*gamm* function) in the *mgcv* R package [73].

## Results

Woody wetlands (52.2%) and upland forests (40.4%) collectively dominated the NNWR, while the remaining land cover classes (e.g. water, developed) only comprised 7.4% of the landscape. Relocations were collected from adult female wild pigs originally captured among 6 trap locations distributed across the NNWR (Fig. 1a) and ranged from 370 to 2317 relocations per wild pig (mean: 1208 ± 224; Fig. 1b). Utilization distributions were highly variable among wild pigs, with core space use ranging from 0.2 to 3.1 km^2^ (mean: 1.2 ± 0.3 km^2^) and total space use ranging from 1.0 to 14.6 km^2^ (mean: 5.2 ± 1.5 km^2^; Table 1). Isopleth values used to identify cores ranged from 48 to 52% (mean: 49.4% ± 0.4%; Table 1).

**Table 1:** Autocorrelated kernel density estimates for two utilization distribution isopleths (core and 90%) for adult female wild pigs (*Sus scrofa*; *n* = 9) trapped in the Sam D. Hamilton Noxubee National Wildlife Refuge (NNWR) in Mississippi, USA

			Autocorrelated Kernel Density Estimate		
Pig ID	Period	Relocations (2-h)	Core Isopleth	Core (km^2^)	90% (km^2^)
**26,619**	09/14/21–12/02/21	370	52%	3.1	14.6
**26,620**	12/02/20–07/08/21	2317	49%	1.3	5.1
**26,626**	08/28/21–02/07/22	1541	49%	2.5	9.1
**26,628**	07/29/21–01/30/22	2186	51%	0.5	2.2
**30,252**	08/28/21–11/17/21	862	48%	0.4	1.4
**35,490**	03/08/21–10/07/21	1158	48%	1.3	4.9
**35,492**	08/12/21–11/18/21	910	50%	0.2	1.0
**35,493**	08/08/21–12/27/21	785	49%	1.4	6.4
**35,494**	11/25/20–02/26/21	746	49%	0.5	2.1

Most wild pigs showed selection for areas located closer to perennial streams (*n* = 8/9 wild pigs), herbaceous and woody wetlands (*n* = 7/9 wild pigs for each) and fields (*n* = 6/9 wild pigs; Table 2). There was also a tendency across individuals for selection of areas located greater distances from upland forest (*n* = 8/9 wild pigs) and open water (*n* = 6/9 wild pigs; Table 2). Absolute selection of developed, shrub, and intermittent streams was generally weaker (Table 2), although realized individual space use was highly variable across the NNWR landscape and likely reflected heterogeneities among these less prominent landscape features within specific areas in which wild pigs were located. However, when individual selection tendencies were applied to utilization distributions (Fig. 1c), hotspots of use were generally localized to fields, woody wetlands, and herbaceous cover near streams. The population-level realized solution showed similar patterns to those seen in individual utilization distributions across the entire NNWR landscape with most predicted use within woody wetland corridors at the center of the NNWR and the least amount of use within upland forests throughout the southwestern NNWR (Fig. 1d).

**Table 2:** Beta (β) coefficients derived from conditional logistic regression models with elastic net penalization (“lasso”) for adult female wild pigs (*Sus scrofa*; *n* = 9) trapped in the Sam D. Hamilton Noxubee National Wildlife Refuge (NNWR) in Mississippi, USA

Pig ID	β_Intermittent_	β_Perennial_	β_Developed_	β_Field_	β_Herbaceous_	β_Shrub_	β_UpFor_	β_Water_	β_WWet_
**26,619**	-	−0.73	−0.02	-	-	-	0.09	0.42	−0.26
**26,620**	−0.21	−0.37	-	0.28	−1.66	0.35	0.51	−0.31	−0.17
**26,626**	0.25	−0.10	−0.24	0.07	−0.16	−0.90	0.56	0.50	0.12
**26,628**	−0.34	1.35	0.62	−0.38	−0.65	0.69	0.07	0.37	−0.07
**30,252**	0.66	−1.24	−0.52	−0.34	1.32	1.87	−0.40	-	−1.01
**35,490**	0.74	−1.32	−0.35	−0.14	−0.18	−0.18	0.33	1.32	−1.79
**35,492**	0.38	−2.18	−1.98	−0.88	−1.79	0.04	1.17	4.57	−0.45
**35,493**	−0.36	−0.37	0.24	−0.29	−0.57	-	0.11	0.20	−0.27
**35,494**	0.04	−1.48	0.80	−0.86	−0.81	-	0.54	−0.93	0.58
**Mean**	0.15	−0.72	−0.18	−0.32	−0.56	0.31	0.33	0.77	−0.37

Finally, daily movements increased from the pre-archery to archery seasons (i.e. Julian days 244–323), before eventually appearing to plateau during the late archery season (i.e. Julian day ∼ 310; Fig. 2a). Movements then declined during the firearms season (i.e. Julian days 324–366; Fig. 2a). Daily movement distances during the pre-archery, archery, and firearms seasons averaged 2152 ± 128 m, 3117 ± 136 m, and 2887 ± 83 m, respectively (Additional File S1: Fig. 3). While general similarities in hourly movement patterns (i.e. crepuscular peaks with diurnal valleys) were apparent among pre-archery, archery, and firearms seasons, movement rates during daylight hours declined during the archery and firearms seasons (Fig. 2b and d).

## Discussion

Estimates of wild pig total space use in this type of landscape are larger than within coastal marshlands (i.e. 1.2 km^2^ [50]), but smaller than estimates reported in other landscapes (e.g. shrubland-grassland; 10.5 km^2^ [47]). Our estimates of core space use were delineated using measures specific to each individual; however, all were comparable to the standard 50% isopleth used in other studies to delineate core use [74, 75], offering general reinforcement to this threshold being used to delineate a core area. Overall, our estimates of space use were comparable to other studies in similar landscapes, which provides support for the scale at which to implement control [76]. It is notable however, that optimal periods for such control may vary by individual or sounder, especially in the southeastern United States which is characterized by temporal inconsistencies in breeding and rearing of offspring, a factor that may contribute to the destabilization of space use, philopatry, and site fidelity [44]. Because of this, alternatives that are fixed in time relative to the timing of the planned control effort (e.g. measures of occurrence; Evans et al., unpublished data) warrant consideration, especially given varying resource availabilities that are likely to influence wild pig movement and occur across large landscapes.

When examining wild pig space use in relation to land cover and streams, we identified general consistencies in selection tendencies across individuals. These tendencies, although variable in strength, reinforced known relationships between wild pigs and areas they rely on for meeting thermoregulatory and foraging requirements [77]. However, despite general underlying homogeneities of the NNWR landscape (i.e. approximately half of the NNWR consisted of contiguous woody wetlands), less prominent features (i.e. herbaceous, fields, and perennial streams) were more sparsely and heterogeneously distributed, and as such were highly variable relative to their availability to individual wild pigs. Considering the life history of wild pigs as generalist and highly adaptive omnivores, the ability for certain individuals to take advantage of areas that are underrepresented or even potentially suboptimal is not surprising. It is also likely that social dynamics (i.e. territoriality) also influence how individuals and their sounders differentially use areas [76].

Disproportionate selection of areas characterized by woody wetlands and streams is common given the need for wild pigs to thermoregulate using available water sources [42, 78], which also supports the lack of use of upland forests as these areas may be associated with fewer thermoregulatory and foraging resources required by wild pigs [28, 29]. While the strength of relationships to woody wetlands was noticeably weaker compared to perennial streams, this was likely due to the pervasiveness of woody wetlands within areas where wild pigs were located. Such abundances in availability often influence the outcome of selection analyses [49], and understanding the role of selection order (as described in [21]) remains imperative, especially when working within generally homogeneous landscapes in which wild pigs position themselves to meet foraging and thermoregulatory requirements. There is also support for wild pig use of herbaceous areas, as this evidence has focused on damage to herbaceous vegetation in otherwise forested areas in northern climates [31] and general selection patterns in the southeastern United States [79]. Given the apparent importance of herbaceous landscapes, such as herbaceous wetlands, to wild pigs, their limited spatial representation in many hardwood forests suggest they could be important areas for damage monitoring, even if difficult to access by human observers. In addition, these areas harbor not only wild pigs, but also floral and faunal species that are likely ecologically sensitive [80], thereby representing areas requiring greater attention when monitoring wild pig damage and informing control efforts when accessible.

We recognize that our sample size of GPS collared pigs was relatively small with slipped collars and harvest pressure making it difficult to collect data for the life of the collar. Despite this, statistically, our sample size was likely more than adequate to describe wild pig preferences [81]. Additionally, the preferences documented herein align with documented preferences in other populations given thermoregulatory and nutritional needs [28, 29, 42, 78] augmenting our confidence in our results. Additionally, when we examined wild pig circadian patterns in relation to anthropogenic pressures in the NNWR, daily distances traveled increased as the seasons progressed from fall into winter. Space use can increase as cooling temperatures make the landscape more thermoneutral to wild pigs or in relation to the emergence of hard mast including acorns (*Quercus* spp.) and hickory nuts (*Carya* spp [47]), making it difficult to infer which of these, seasonality or hunting pressure, was the cause for changes in circadian rhythm at a seasonal scale. However, it is notable that daily distances plateaued and then diminished during the firearms season which is typical for wild pigs that may experience increased anthropogenic pressures associated with hunting seasons [39, 71]. This pattern is further exemplified when we examine daily circadian rhythms as there were increasingly restricted diurnal movements, a trend that is not unexpected on public lands which are also subjected to frequent anthropogenic pressures during open hunting seasons [39, 72]. Although general trends were similar and reflective of the life history of wild pigs (i.e. primarily crepuscular [47]), the decreases in diurnal movement distances during the archery and firearms seasons indicate that wild pigs are responding to anthropogenic pressures, even within this landscape which provides many natural refuges (e.g. inaccessible wetlands) and contains a low density of wild pigs given the relatively recent invasion of this landscape. Therefore, natural resource managers must understand wild pigs have spatiotemporal complexities (e.g. restriction of movement to only certain hours within certain landscapes) that should be considered when attempting to implement control measures such as trapping, especially during periods which are characterized by movement shifts related to anthropogenic activities, as these will likely impact efficiency and effectiveness of these measures.

## Conclusions

While our investigation provides general reinforcement to earlier findings on wild pig space use in other regions, it also identified the need to investigate movement phenomena from various angles among multiple spatiotemporal scales. Seemingly, wild pigs are very flexible in their movement patterns and resource use, exhibiting individuality that may reflect generalist tendencies, and changes within individuals relative to spatiotemporally dependent external drivers further compound the challenges faced by resource managers. Thus, it is imperative that movement patterns be characterized across not only individuals but also the breadth of landscapes they can invade, and further consideration should be given to the social interactions that are also occurring within these intensively used areas [82], as this information will be critical to developing substantive and meaningful control efforts. Collectively, our findings will add to the knowledge required by natural resource managers to both control wild pigs and protect our native natural resources.

### Electronic supplementary material

Below is the link to the electronic supplementary material.

Supplementary Material 1

## Data Availability

The dataset supporting the conclusions of this article will be made available in the Dryad Digital Repository.
