# Supplementary material for: What drives wild pig (Sus scrofa) movement in bottomland and upland forests?
Source: Mov Ecol. 2024 Apr 25;12:32. doi: 10.1186/s40462-024-00472-y (PMC11044336; doi:10.1186/s40462-024-00472-y)
Supplement: Supplementary file 1 — Supplementary Material 1 [file 40462_2024_472_MOESM1_ESM.docx]

**Additional File 1**


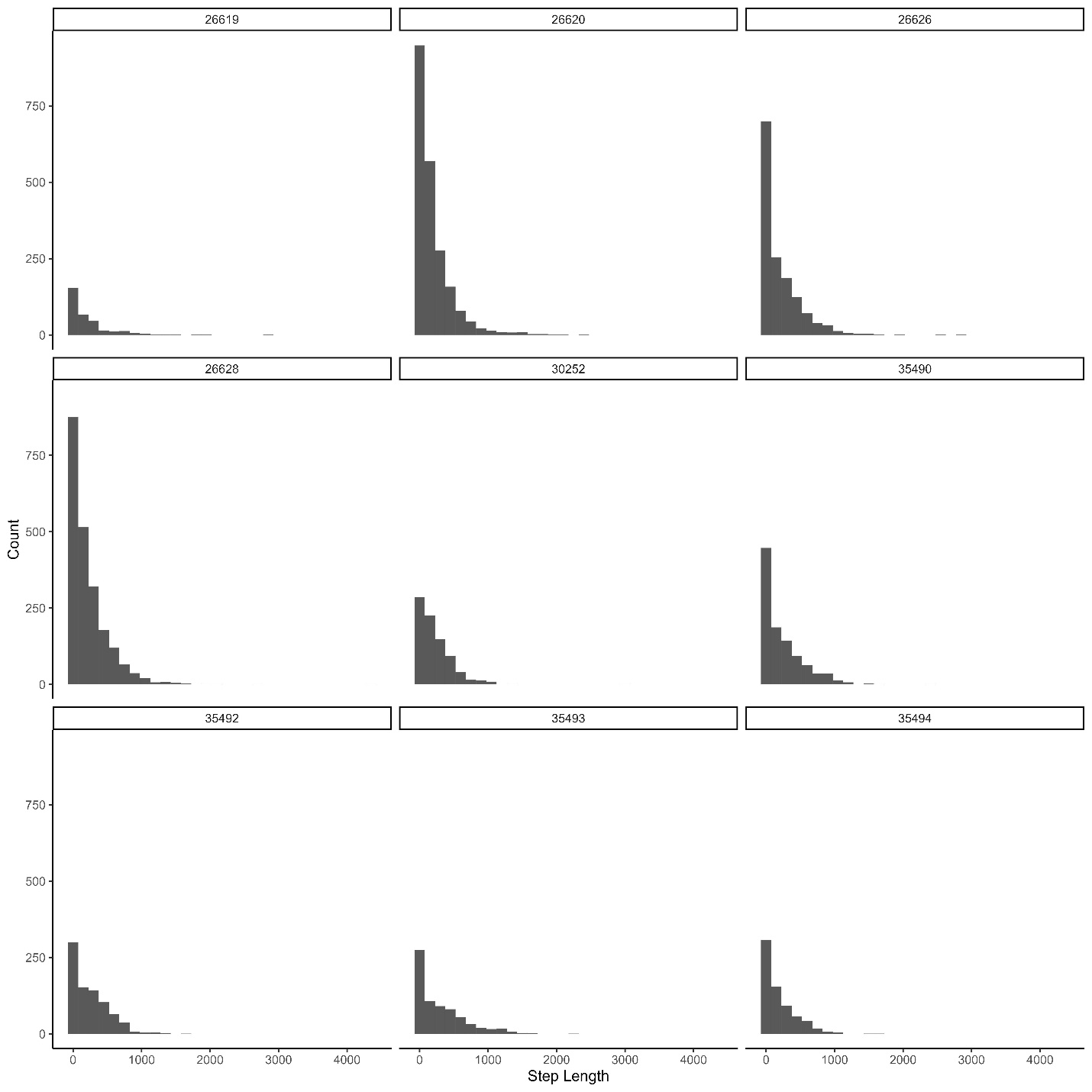
**Figure 1.** Distribution of step lengths (m) for adult female wild pigs (*Sus scrofa*; *n* = 9) trapped in the Sam D. Hamilton Noxubee National Wildlife Refuge (NNWR) in Mississippi, USA.


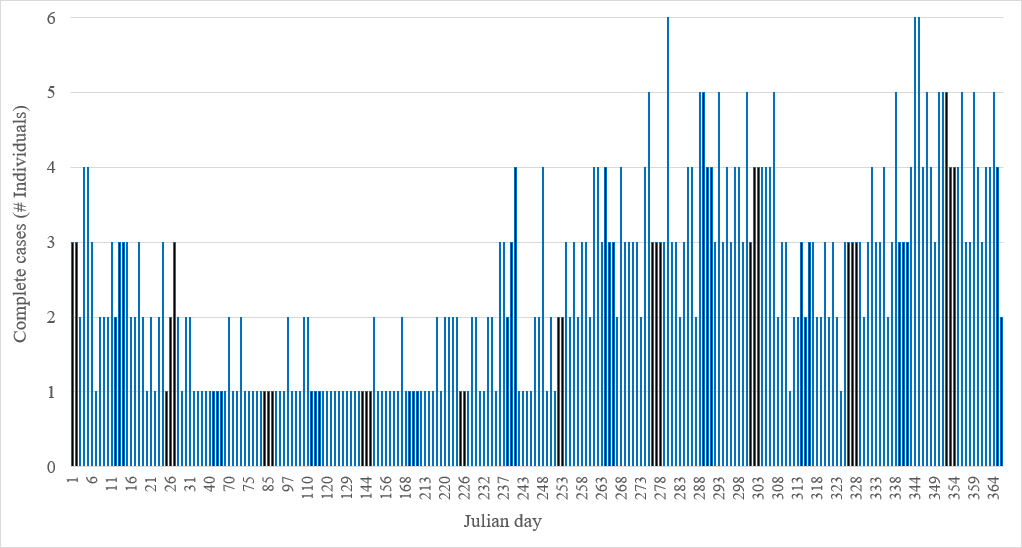
**Figure 2.** Number of complete cases (*n* = 12 relocations/day) relative to Julian day for adult female wild pigs (*Sus scrofa*) trapped in the Sam D. Hamilton Noxubee National Wildlife Refuge (NNWR) in Mississippi, USA.**
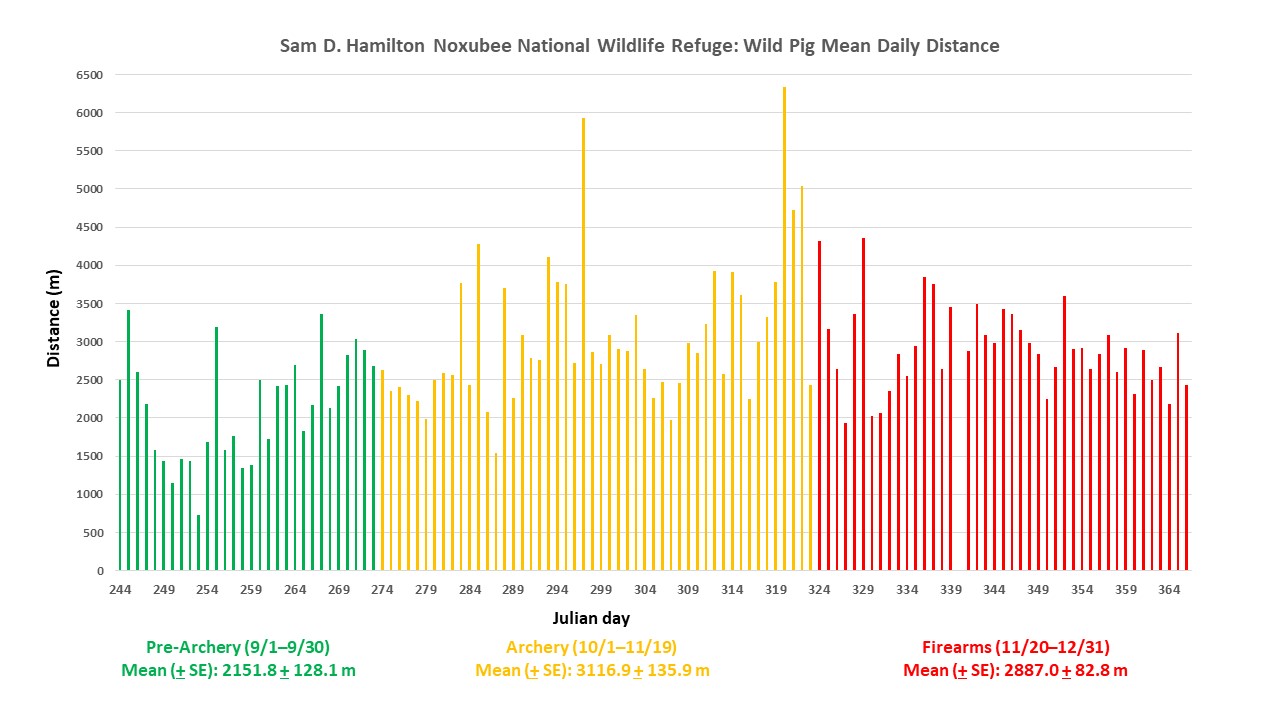
**

**Figure 3.** Mean daily distances traveled by wild pigs (*Sus scrofa*) relative to Julian day during the pre-archery, archery, and firearms seasons in the Sam D. Hamilton Noxubee National Wildlife Refuge (NNWR) in Mississippi, USA.
